# Chloroquine Clinical Failures in *P. falciparum* Malaria Are Associated with Mutant *Pfmdr-1*, Not *Pfcrt* in Madagascar

**DOI:** 10.1371/journal.pone.0013281

**Published:** 2010-10-13

**Authors:** Valérie Andriantsoanirina, Arsène Ratsimbasoa, Christiane Bouchier, Magali Tichit, Martial Jahevitra, Stéphane Rabearimanana, Rogelin Raherinjafy, Odile Mercereau-Puijalon, Rémy Durand, Didier Ménard

**Affiliations:** 1 Institut Pasteur, Antananarivo, Madagascar; 2 Ministère de la Santé, du Planning Familial et de la Protection Sociale, Antananarivo, Madagascar; 3 Institut Pasteur, Paris, France; 4 Hôpital Avicenne, AP-HP, Bobigny, France; Université Pierre et Marie Curie, France

## Abstract

Molecular studies have demonstrated that mutations in the *Plasmodium falciparum* chloroquine resistance transporter gene (*Pfcrt*) play a major role in chloroquine resistance, while mutations in *P. falciparum* multidrug resistance gene (*Pfmdr-1*) act as modulator. In Madagascar, the high rate of chloroquine treatment failure (44%) appears disconnected from the overall level of *in vitro* CQ susceptibility (prevalence of CQ-resistant parasites <5%) or *Pfcrt* mutant isolates (<1%), strongly contrasting with sub-Saharan African countries. Previous studies showed a high frequency of *Pfmdr-1* mutant parasites (>60% of isolates), but did not explore their association with *P. falciparum* chloroquine resistance. To document the association of *Pfmdr-1* alleles with chloroquine resistance in Madagascar, 249 *P. falciparum* samples collected from patients enrolled in a chloroquine *in vivo* efficacy study were genotyped in *Pfcrt*/*Pfmdr-1* genes as well as the estimation of the *Pfmdr-1* copy number. Except 2 isolates, all samples displayed a wild-type *Pfcrt* allele without *Pfmdr-1* amplification. Chloroquine treatment failures were significantly associated with *Pfmdr-1* 86Y mutant codon (OR = 4.6). The cumulative incidence of recurrence of patients carrying the *Pfmdr-1* 86Y mutation at day 0 (21 days) was shorter than patients carrying *Pfmdr-1* 86N wild type codon (28 days). In an independent set of 90 selected isolates, *in vitro* susceptibility to chloroquine was not associated with *Pfmdr-1* polymorphisms. Analysis of two microsatellites flanking *Pfmdr-1* allele showed that mutations occurred on multiple genetic backgrounds. In Madagascar, *Pfmdr-1* polymorphism is associated with late chloroquine clinical failures and unrelated with *in vitro* susceptibility or *Pfcrt* genotype. These results highlight the limits of the current *in vitro* tests routinely used to monitor CQ drug resistance in this unique context. Gaining insight about the mechanisms that regulate polymorphism in *Pfmdr1* remains important, particularly regarding the evolution and spread of *Pfmdr-1* alleles in *P. falciparum* populations under changing drug pressure which may have important consequences in terms of antimalarial use management.

## Introduction

*Plasmodium falciparum* resistance to chloroquine (CQ) has emerged at least from six independent foci (South East Asia, Venezuela, Colombia, Papua New Guinea, India and Philippines) in the late 1950s and in the 1960s [1], [2], [3]. Molecular evolutionary studies have demonstrated that *P. falciparum* CQ-resistant parasites from South-East Asia have entered in East Africa (Kenya and Tanzania) in the late 1970s and spread across the African continent within two decades [2]. *P. falciparum*-related deaths rose sharply after the spread of CQ-resistant parasites in sub-Saharan Africa, affecting mostly children under 5 years of age [4], [5], [6].

CQ-resistance in *P. falciparum* shows some biological similarities with the multiple drug resistance phenotype of mammalian tumour cells, as both involve expulsion of drug out of the cytosol of the cell and can be reversed by calcium channel antagonists such as verapamil [7]. Based on molecular allele exchange studies and analysis of genetic crosses, it is today generally accepted that the major role in CQ resistance is determined by polymorphisms in *Pfcrt*, a gene encoding a transporter which promotes, in its mutated forms, drug efflux from the parasite digestive vacuole, while *Pfmdr-1* modulates the level of *in vitro* CQ-resistance [3], [8], [9]. In addition, mutations or amplifications of *Pfmdr-1* gene can play a significant role in *P. falciparum* resistance to diverse antimalarials such as mefloquine, quinine or artemisinin derivatives [10]. Specific combinations of *Pfcrt* and *Pfmdr-1* alleles, resulting in varying responses to CQ (and amodiaquine), appeared geographically restricted, which may explain why some field studies reported an association between *Pfmdr-1* polymorphisms and CQ resistance and other studies did not [9].

In Madagascar, CQ was first introduced in 1945 and was largely used during more than 50 years, in particular in a campaign for malaria prevention treatment in children [11]. A shortage in CQ supply in the 1970s was followed by a large-scale epidemic in the late 1980s. Wide-scale access to CQ treatment resumed afterwards, and CQ is still used today though its replacement by the artesunate plus amodiaquine combination therapy is recommended as first-line treatment for uncomplicated cases since 2005. The first clinical cases of CQ resistance were suspected in 1975. In 1981, other cases of treatment failures were reported and *in vitro* tests showed isolates with high 50% inhibitory concentration for CQ (IC_50_) indicative of *in vitro* CQ-resistance. However, during the 1981-2008 period, several studies outlined lower rates of *in vitro* CQ-resistance and therapeutic failures in Madagascar than in other African countries [11]. Currently, the prevalence of *in vitro* CQ resistance does not exceed 5% of isolates and *Pfcrt* mutant parasites are found in less than 1% of collected isolates [12]. Surprisingly, these figures appear disconnected from the high level of clinical treatment failures (44%) [13], most of which (∼90%) are late clinical/parasitological treatment failures. Interestingly, previous surveys outlined the presence of *Pfmdr-1* mutations in more than 60% of isolates, suggesting that *Pfmdr-1* mutants could be responsible for CQ clinical failure in Madagascar.

The aim of the present work was to document the association of *Pfmdr-1* with *in vivo* CQ clinical failure and *in vitro* CQ resistance. To this end, two independent sets of *P. falciparum* samples collected in 2006–2007 from *in vivo* and *in vitro* susceptibility studies were genotyped for *Pfcrt* and *Pfmdr1* genes as well as the estimation of the *Pfmdr-1* copy number. Isolates that meet the inclusion criteria were included in the association analysis (240 from *in vivo* study and 90 from *in vitro* testing). In addition, the evolutionary dynamics of the *Pfmdr-1* locus in the Malagasy parasite populations were also assessed. Our data show that in this particular setting, *Pfmdr-1* polymorphism on multiple genetic backgrounds, in absence of mutations in *Pfcrt* gene or *Pfmdr-1* gene amplification was significantly associated with late chloroquine clinical failures and unrelated with the overall level of *in vitro* CQ susceptibility, raising the limits of the current *in vitro* tests routinely used to monitor CQ drug resistance.

## Materials and Methods

### Study Sites

*P. falciparum* samples from eight sentinel sites involved in the monitoring of the antimalarial drug resistance in Madagascar [12] were studied. The collection sites were located in the four main malarious epidemiological strata: Ejeda and Ihosy in the South (sub-desert stratum, epidemic prone), Maevatanana and Miandrivazo in the West (tropical stratum, seasonal and endemic area), Tsiroanomandidy and Moramanga in the foothills of the Central Highlands (highlands stratum, low-endemic area) and Farafangana and Andapa in the East (equatorial stratum, perennial endemic area). Malaria transmission, estimated by annual entomological inoculation rate (EIR, number of bites of infected anophelines per person sleeping indoors), varied according to the sites from 0.2 (in the South) to 240 (in the East) [14]. The prevalence of anti-MSP1 antibodies used as proxy of the burden of malaria in each site was previously determined at the same collection period [15] and were included in our analysis.

### Patients and *in vivo* study

In 2006–2007, multi-site prospective chemotherapeutic studies were carried out for assessing the therapeutic efficacy of antimalarial therapies recommended by the National Malaria Control Programme (NMCP) (registration number ISRCTN36517335) [13]. Patients with microscopically confirmed uncomplicated *P. falciparum* malaria were randomized into one of the four treatment groups: Chloroquine (CQ), Amodiaquine (AQ), Sulfadoxine-Pyrimethamine association (SP) and Artesunate-Amodiaquine combination (ASAQ). According to the 2003 WHO protocol, patients aged from six months to 15 years, presenting with *P. falciparum* monoinfection (parasitaemia from 1,000 to 200,000/µL), axillary temperature ≥37.5°C, body weight >5 kg, haemoglobin (Hb) ≥5 g/dL, without severe malnutrition, signs of severity, or a concomitant disease were included in the clinical trial. Once written informed consent was given by the patient or the guardian and after randomization, patients were administered either CQ (10 mg/kg on days 0 and 1, and 5 mg/kg on day 2), AQ (10 mg/kg on days 0, 1, and 2), SP (25 mg/kg sulfadoxine and 1.25 mg/kg pyrimethamine as a single dose on day 0) or ASAQ (AS: 4 mg/kg on days 0, 1, and 2 and AQ:10 mg/kg on days 0, 1, and 2) and seen on days 1, 2, 3, 7, 14, 21 and 28, or any intervening day if they were unwell for malaria infection to assess the clinical and parasitological efficacy of the drug regimens. Blood samples were obtained by finger prick on the enrolment and all follow-up days including any unscheduled day to use for analysis of thick and thin blood smears and for storage on filter paper for further molecular analysis. Haemoglobin concentration was measured on day 0 using a HemoCue haemoglobinometre (HemoCue AB, Ängelholm, Sweden).

### Collection of *P. falciparum* isolates for *in vitro* drug sensitivity testing

As part of the surveillance of antimalarial drug resistance in Madagascar, fresh *P. falciparum* clinical isolates were routinely collected in 2006–2007 from symptomatic patients prior to treatment in six of the eight sites (Ihosy, Maevatanana, Miandrivazo, Tsiroanomandidy, Moramanga and Farafangana). Venous blood samples were collected in tubes coated with EDTA (Vacutainer tubes, Becton Dickinson, Rutherford, NJ, USA), from malaria-positive patients (>2 years old) after they gave their consent for participation in the study (approval number 007/SANPF/2007). Presence of malaria parasites was evaluated by using a rapid diagnostic test based on the detection of *Plasmodium*-specific lactate dehydrogenase (pLDH) (OptiMAL-IT, DiaMed AG, Cressier sur Morat, Switzerland). Positive patients were treated with the artesunate-amodiaquine combination, according to the National Malaria Control Programme (NMCP), and samples were sent to the Malaria Research Unit of the Institut Pasteur of Madagascar in a controlled cool box at 4°C. Drug sensitivity assays were done using the classical isotopic 48-h test, as described [12], on blood samples with parasitaemia ≥0.1%, available within 48 h after blood collection and from patients declaring no antimalarial drug intake during the previous 7 days. For CQ sensitivity assay, three control wells without drug were used as control and each concentration ranging from 12.5 to 1,600 nM was studied in duplicate or triplicate. The IC_50_, i.e. the drug concentration corresponding to 50% of ^3^H-hypoxanthine uptake by the parasites in drug-free control wells, was determined by probit/logit regression analysis. The quality of the assays was controlled by using *P. falciparum* reference lines (3D7 Africa, CQ-sensitive clone and FCM29 Cameroon, CQ-resistant clone).

### DNA extraction

Parasite DNA was available from whole blood or capillary blood transferred to filter paper (Whatman, Maidstone, UK). DNA was extracted from blood spots with Instagene® Matrix resin (BioRad©, Marnes la Coquette, France), according to the manufacturer's instructions or directly from 100 µl of whole blood by using the phenol-chloroform method [16]. Parasite species was confirmed by using real-time polymerase chain reaction as described by de Monbrison *et al.* [17].

### *Pfmdr-1* and *Pfcrt* genotyping in isolates from day 0 and day of recurrence

*Pfmdr-1* and *Pfcrt* genes were amplified in samples from enrolment (and day of recurrence in cases of CQ treatment failure) by nested PCR approach, as described [12]. For *Pfmdr-1* gene, two fragments (549 bp, from codons 23 to 206 and 938 bp, from codons 966 to 1278) and for *Pfcrt* gene, three fragments (450 bp, from codon 37 in exon 2 to codon 125 in exon 3, 447 bp, from codon 121 in exon 3 to codon 211 in exon 4 and 850 bp, from codon 199 in exon 3 to codon 316 in exon 7) were sequenced. Sequencing reactions were carried out with ABI Prism BigDye Terminator cycle sequencing ready reaction kit and were run on a model 3730 xl genetic analyzer (Applied Biosystems, Courtaboeuf, France). Electrophoregrams were visualized and analyzed with CEQ2000 genetic analysis system software (Beckman Coulter, Villepinte, France) and amino acid sequences were compared with the wild-type amino acid sequences (GenBank accession numbers, AF030694 for *Pfcrt*, and XM_001351751 for *Pfmdr-1*). The presence of single nucleotide polymorphisms (SNPs) was confirmed by reading both strands. Sequences of insufficient quality were either resequenced or rejected. *Pfmdr-1* and *Pfcrt* alleles were reconstructed from full sequences presenting an unambiguous single allele signal at each nucleotide position.

### *Pfmdr-1* copy number determination

The *Pfmdr-1* copy number was measured by TaqMan real-time PCR (Rotor-Gene 6000, Corbett Research, Sydney, Australia) relative to the single copy of the ß-tubulin (used as a housekeeping gene), as described [12]. All samples were run in duplicate in 25 µL reaction mixtures. For each run, the *Pfmdr-1* copy number was measured relative to the numbers in two standard calibrator parasite clonal lines (3D7 Africa and Dd2), by the ΔΔCT method [18]. Reference DNA clone W2 (which has three copies of *Pfmdr-1*) was used as the quality control in each run.

### Analysis microsatellite flanking *Pfmdr-1*

Two microsatellite markers (MS loci 956456 and 957861), located on chromosome 5 and extending ∼10 kb downstream the *Pfmdr-1* gene were used to determine the evolutionary history of *Pfmdr-1* alleles. Microsatellite polymorphism was analyzed using a nested PCR strategy, as previously described by Mehlotra et al. [9]. Microsatellite PCR products were genotyped on the basis of size, using a GeneScan 500 LIZ size standard on an ABI Prism 3730 XL DNA analyzer.

### Assessment of isolate clonality

The number of genotypes present in isolates collected from *in vivo* and *in vitro* assays, was estimated by using an allelic family-specific nested PCR (MAD20, K1, and RO33 for *Pfmsp-1* and 3D7 Africa and FC27 for *Pfmsp-2*) [12]. Clonality was defined as the highest number of alleles detected at either of the two loci and used to classify isolates as monoclonal or polyclonal and to distinguish recrudescence from new infection for all patients failing therapy after day 7 (isolates from day 0 and day of recurrence). All PCR amplifications contained a positive control (genomic DNA from strains W2, HB3, and 3D7 Africa) and a negative control (no target DNA).

### Statistical analysis

Data were entered and verified using Microsoft Excel^©^ software, and analyzed using EpiInfo 6.04^©^ software (Centers for Disease Control and Prevention, Atlanta, GA, United States) and XLSTAT^©^ for Windows XP (Addinsoft, Paris, France). For *in vivo* clinical trial samples, criteria of inclusion for the analysis were (i) patients with complete 28-days follow-up, (ii) absence of reinfection, (iii) wild-type *Pfcrt* alleles in isolates collected at day 0 and day of recurrence and (iv) successful determination of *Pfmdr-1* alleles in isolates from day 0 and day of recurrence. For *in vitro* testing samples, criteria of inclusion for the analysis were (i) monoclonal isolates by using *msp1/msp2* genotyping, (ii) wild-type *Pfcrt* alleles and (iii) successful determination of *Pfmdr-1* alleles.

The Mann-Whitney U test or Kruskal-Wallis method were used for non-parametric comparisons, and Student's t test or one-way analysis of variance for parametric comparisons. For categorical variables, Chi-squared or Fisher's exact tests were used to assess significant differences in proportions. Odds ratios (OR) and their 95% confidence intervals (95%CI), describing the association between CQ clinical outcomes and exposure variables were determined by conditional logistic regression.

For the multivariate analysis, variables with *P*-values <0.25 were initially introduced into the model and removed following a backwards-stepwise selection procedure to leave only those with a P-value <0.05 in the final model. The relation between *Pfmdr-1* 86Y mutation in isolates from day of recurrence and clinical response to CQ treatment was assessed by survival analysis using the Kaplan-Meier method and the log-rank test. The median asexual parasite densities clearance time (in percent of value on day 0) was also compared between patients according to the *Pfmdr-1* alleles (86N and 86Y) found at day 0 (and day of recurrence). All reported *P*-values are two-sided and were considered statistically significant if less than 0.05.

Genetic diversity was assessed by Nei's unbiased expected heterozygosity (He) from haploid data and calculated as H_e_ = [n/(n−1)][1−p_i_] (n = the number of isolates sampled; p_i_ = the frequency of the i^th^ allele [19]. Population genetic differentiation was measured using Wright's F statistics [20]; population genetic parameters were computed with FSTAT software, v2.9.4 [21].

### Ethical approval

The study protocol was reviewed and approved by the Ethics Committee of the Ministry of Health of Madagascar (approval number 007/SANPF/2007; registration number ISRCTN36517335). Informed written consent was provided by all patients or their parents/guardians before inclusion in the study.

## Results

### *In vivo* and *in vitro* sample collection

**Figure 1:**
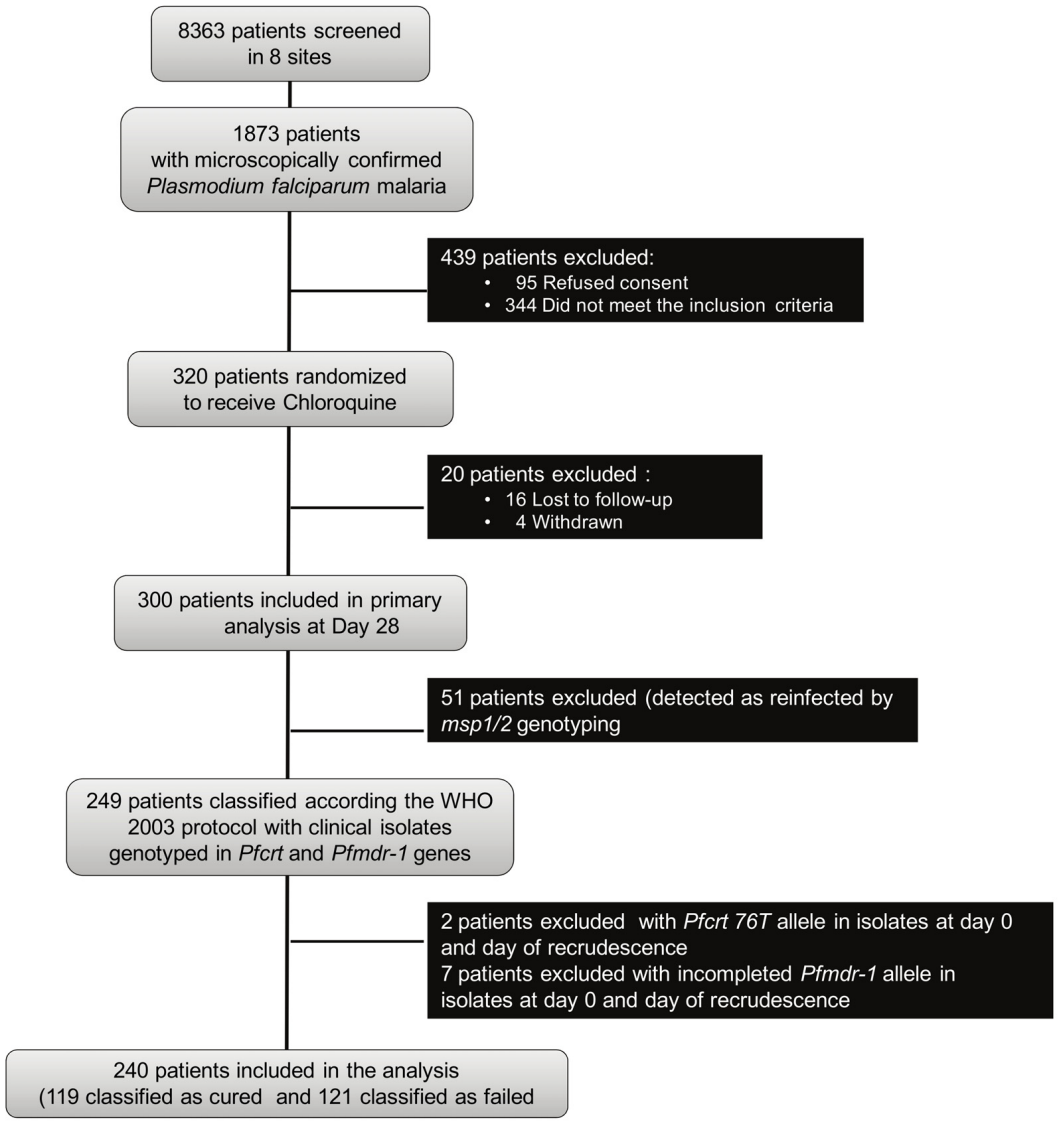
Flowchart of patients. Flowchart of patients: Enrollment, Randomization, Follow-up, Molecular genotyping.

In the *in vivo* clinical trial, a total of 8363 febrile children attending in health centres from the sentinel sites were screened for *falciparum* malaria and 1873 were microscopically positive for *P. falciparum*. They were assigned in different treatment groups. Among the 320 patients treated with CQ, 240 of them (75%) met the study analysis criteria. According to the WHO 2003 protocol, 119 patients were classified as cured and 121 as failing treatment (13 patients failed before day 7, 20 patients between days 8–14, 46 patients between days 15–21 and 42 patients between days 22–28). Late treatment failures accounted for 89.3% of the overall treatment failures. Details are given in figure 1.

Among the 420 *P. falciparum* isolates tested for *in vitro* susceptibility to CQ, 372 (88.6%) were successfully assayed. All isolates presented a wild type *Pfcrt* allele; ninety of them, meeting the study analysis criteria, were included in the analysis. Among the 90 isolates *in vitro* tested, the geometric mean and the median of CQ IC_50_ was 18.7 nM (95%CI 14.7–23.7 nM) and 22 nM (95%CI 16.2–25.0 nM), respectively.

### *Pfmdr-1* alleles, copy number of *Pfmdr-1* gene

*Pfmdr-1* alleles were determined for the 240 isolates from the *in vivo* efficacy study (day 0 and day of recurrence, when available) and for 90 isolates for which the IC_50_ for CQ was determined. Amongst *in vivo* isolates, three of five major SNPs previously related to CQ-resistance were observed at day 0, N86**Y** (147/240, 61.2%), Y184**F** (172/240, 71.7%), and D1246**Y** (80/240, 33.3%), present in eight different alleles: the wild-type allele NYD (11.2%), three single-mutant alleles (N**F**D, 22.9%; **Y**YD, 14.6% and NY**Y**, 1.2%), three double-mutant alleles (**YF**D, 17.9%; N**FY**, 3.3% and **Y**Y**Y**, 1.2%) and one triple-mutant allele (**YFY**, 27.5%). Discordant alleles were found in 24 isolates between isolate from day 0 (wild-type NYD) and isolate from day of recurrence (13 single-mutant alleles **Y**YD, 8 single-mutant alleles N**F**D and 3 double-mutant alleles **YF**D). There was a non-random association between the N86Y and D1246Y loci (*R^2^* = 0.23, *P* = 0.004) and F184Y and D1246Y loci (*R^2^* = 0.28, *P* = 0.0003).

Amongst *in vitro* isolates, only three isolates had an IC_50_>100 nM: one triple-mutant allele (**YFY**, IC_50_ = 142 nM), one double-mutant allele (**YF**D, IC_50_ = 126 nM) and one single-mutant allele (N**F**D, IC_50_ = 140 nM). Comparison of geometric mean IC_50_ between isolates harbouring different *Pfmdr-1* alleles showed no significant difference: 18.0 nM (n = 37, 95%CI 12.1–26.6 nM) in *Pfmdr-1* 86N isolates compared to 19.2 nM (n = 53, 95%CI 14.1–26.0 nM) in *Pfmdr-1* 86Y isolates, 14.3 nM (n = 72, 95%CI 8.2–25.0 nM) in *Pfmdr-1* 184Y isolates compared to 20.0 nM (n = 18, 95%CI 15.3–26.1 nM) in *Pfmdr-1* 184F isolates and 21.4 nM (n = 61, 95%CI 13.5–36.7 nM) in *Pfmdr-1* 1246D isolates compared to 18.6 nM (n = 21, 95%CI 12.3–28.3 nM) in *Pfmdr-1* 1246Y isolates.

The *Pfmdr-1* copy number was determined for 290 isolates (219 from *in vivo* studies and 71 from *in vitro* assays). The mean copy number was 0.93 (ranging from 0.74 to 1.33). When the value was rounded to the nearest integer, no *Pfmdr-1* amplification was observed.

### *Pfmdr-1* microsatellite polymorphism

**Figure 2:**
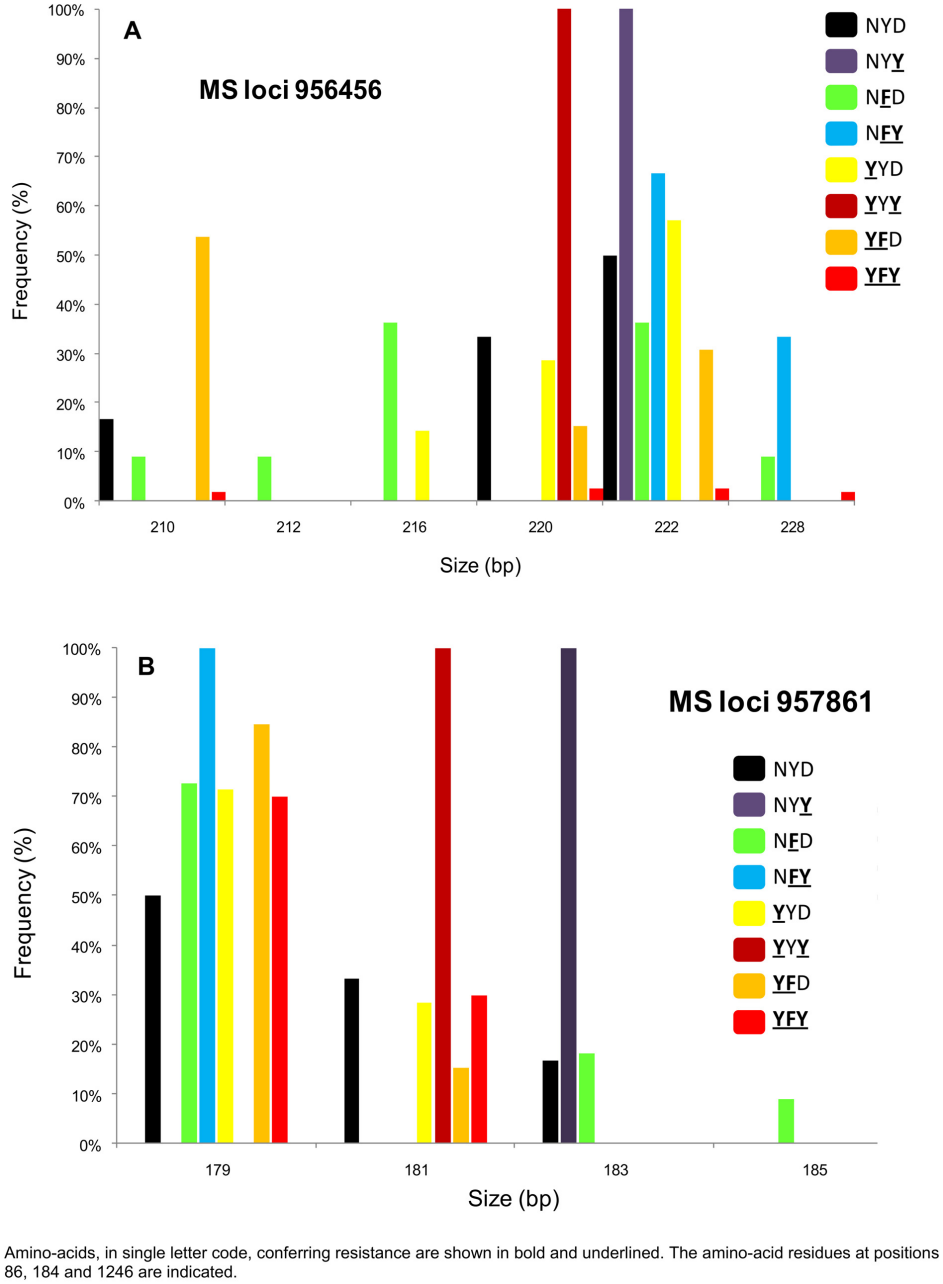
Prevalence of the two microsatellite loci flanking *Pfmdr-1* gene. Distribution and prevalence of the two microsatellite loci flanking *Pfmdr-1* gene (MS 956456, panel A and MS 957861, panel B) in 53 *Plasmodium falciparum* isolates collected from Madagascar in 2006–2007.

The polymorphisms in two microsatellite loci flanking the wild-type coding sequence and mutant-type alleles (single-mutants to triple-mutant) are shown in Figure 2. The MS locus 956456 had 6 alleles (ranging from 210 to 228 bp) and the MS locus 957861 had 4 alleles (ranging from 179 to 185 bp), displaying 7 different haplotypes. Microsatellite markers were moderately polymorphic for wild- and mutant-type alleles with a mean Nei's unbiased expected heterozygosity (He) ranging from 0.26 (N**FY**) to 0.66 (NYD). The Wright's fixation index analysis showed a significant absence of genetic differentiation between allele populations.

### *In vivo* outcomes and risk factors associated to CQ treatment failure

To test whether any collected variables (ecological environment, patients and isolates characteristics) were associated with clinical response to CQ treatment, we compared them between both cured- and failing treatment patient groups (Table 1). Among these, four variables were significantly associated with the risk of CQ treatment failure: (i) prevalence of anti-PfMSP-1 antibodies previously estimated in the site (*P* = 0.04, 1.8-fold increased risk of CQ treatment failures in area of unstable malaria), (ii) age group (*P*<0.0001, increased risk of CQ clinical failures inversely correlated to age: 1.6-fold in the 6–10 years age group and 4.5-fold in the 0.5–5 years age group), (iii) haemoglobin concentration at day 0 (*P*<0.0002, 2.4-fold increased risk of CQ treatment failures for people with haemoglobin concentration <10 g/dL at day 0) and (iv) presence of the *Pfmdr-1* 86Y mutation in isolates collected on day 0 (*P* = 0.009, 2-fold increased risk of CQ treatment failures) or on day of recurrence (*P*<0.0001, 4.2-fold increased risk of CQ treatment failures).

**Table 1:** Univariate (conditional logistic regression) analysis of risks factors associated to CQ-treatment failure (recrudescence), Madagascar, 2006–2007.

Exposure variables		Treatment outcome¥				*P*-value	OR#	95% CI¶
		Failed		Cured				
		n	value	n	value			
**Collection sites**								
Epidemiological strata^1^	Tropical (reference)	51	49.5%	52	50.5%	0.94*	-	-
	Highlands	32	52.5%	29	47.5%		1.1	0.58–2.07
	Sub-desert	38	50.0%	38	50.0%		1.0	0.55–1.80
EIR (number of bites of infected anophelines per person sleeping indoors)^2^	<5 (reference)> = 5	7051	51.1%49.5%	6752	48.9%50.5%	0.89§	-0.95	-0.57–1.60
Prevalence of anti-PfMSP-1 antibodies^3^	<40% (reference)> = 40%	4972	43.4%56.7%	6455	56.6%43.3%	0.04§	-0.57	-0.34–0.96
**Patient characteristics**								
Male gender		121	44.3%	119	50.4%	0.2§	0.78	0.47–1.30
Age	mean (± SD), year	121	4.8 (3.7)	119	7.03 (4.34)	<0.0001†	0.88	0.83–0.94
	0.5–5 (reference)	79	64.8%	43	35.2%	-	-	-
	6–10	33	39.5%	49	60.5%	0.0005*	0.36	0.20–0.63
	11–15	11	28.9%	27	71.1%	0.0002*	0.22	0.10–0.49
Temperature (°C) at day 0. mean (± SD)		121	38.4 (0.7)	119	38.3 (0.8)	0.61†	1.0824	0.80–1.47
Haemoglobin concentration (g/dL) at Day 0.	mean (± SD), g/dL<10> = 10 (reference)	1217349	9.4 (2.4)61.340.1	1194673	10.4 (2.1)38.7%59.9%	0.0002†0.001*-	0.82.36-	0.71–0.901.41–3.96
Previous CQ in-take (%)		121	8.2	119	8.4	0.57*	0.97	0.39–2.43
**Clinical isolates**								
Parasitaemia density at Day 0 (no.of parasites/µL). mean (± SD)		121	41,435 (60,928)	119	43,289 (55,338)	0.8†	1	-
Multiplicity of infection (MOI) mean. (± SD)		121	1.9 (0.9)	119	1.7 (0.8)	0.06†	0.39	0.98–1.96
*Pfmdr*-1 86Y,%	at Day 0	121	67.7%	119	52.9%	0.009§	2.01	1.18–3.42
	at Day of recrudescence±	121	82.6%			<0.0001§	4.23	2.34–7.65
Pfmdr-1 184F,%	at Day 0	121	71.9%	119	71.4%	0.90§	1.02	0.58–1.79
	at Day of recrudescence±±	121	81.0%			0.08§	1.70	0.93–3.11
Pfmdr-1 1246Y. %	at Day 0	121	31.3%	119	31.9%	0.98§	0.99	0.50–3.11

¥ Treatment failure was based on the World Health Organization 28-day drug efficacy test and monitoring and corrected by PCR genotyping as described in Materials ans Method section;

*Chi-squared test;

§ Fisher exact test;

† ANOVA test (or if P-value of the Bartlett's test for inequality of population variances was <0.05, Mann–Whitney/Wilcoxon two-sample tests);

# OR: odds ratio;

¶ 95% Confidence Interval;

± Discordant alleles found in 16 isolates between day 0 (wild-type NYD) and day of recrudescence (13 single-mutants **Y**YD and 3 double-mutants **YF**D);

±± Discordant alleles found in 8 additional isolates between day 0 (wild-type NYD) and day of recrudescence (8 single-mutants N**F**D);

1 according to [18];

2 according to [19] and

3 according to [20].

In the multivariate analysis, variables with *P*-values <0.25 were initially introduced into the model (prevalence of anti-PfMSP-1 antibodies, gender, age, haemoglobin concentration at day 0, multiplicity of infection, presence of *Pfmdr-1* codon mutation at position 86 in isolates from day 0 and day of recurrence and at position 184 in isolates from day of recurrence). Following a backwards-stepwise selection procedure, CQ treatment failures were significantly associated with presence of *Pfmdr-1* codon mutation at position 86 in the parasites collected on day of recrudescence (OR = 4.6, 95%CI 2.3 to 8.9, *P*<0.0001), age (OR = 1.2, 95%CI 1.1 to 1.3, *P* = 0.0002) and prevalence of antiPfMSP-1 antibodies (OR = 1.02, 95%CI 1.0 to 1.05, *P* = 0.02).

**Figure 3:**
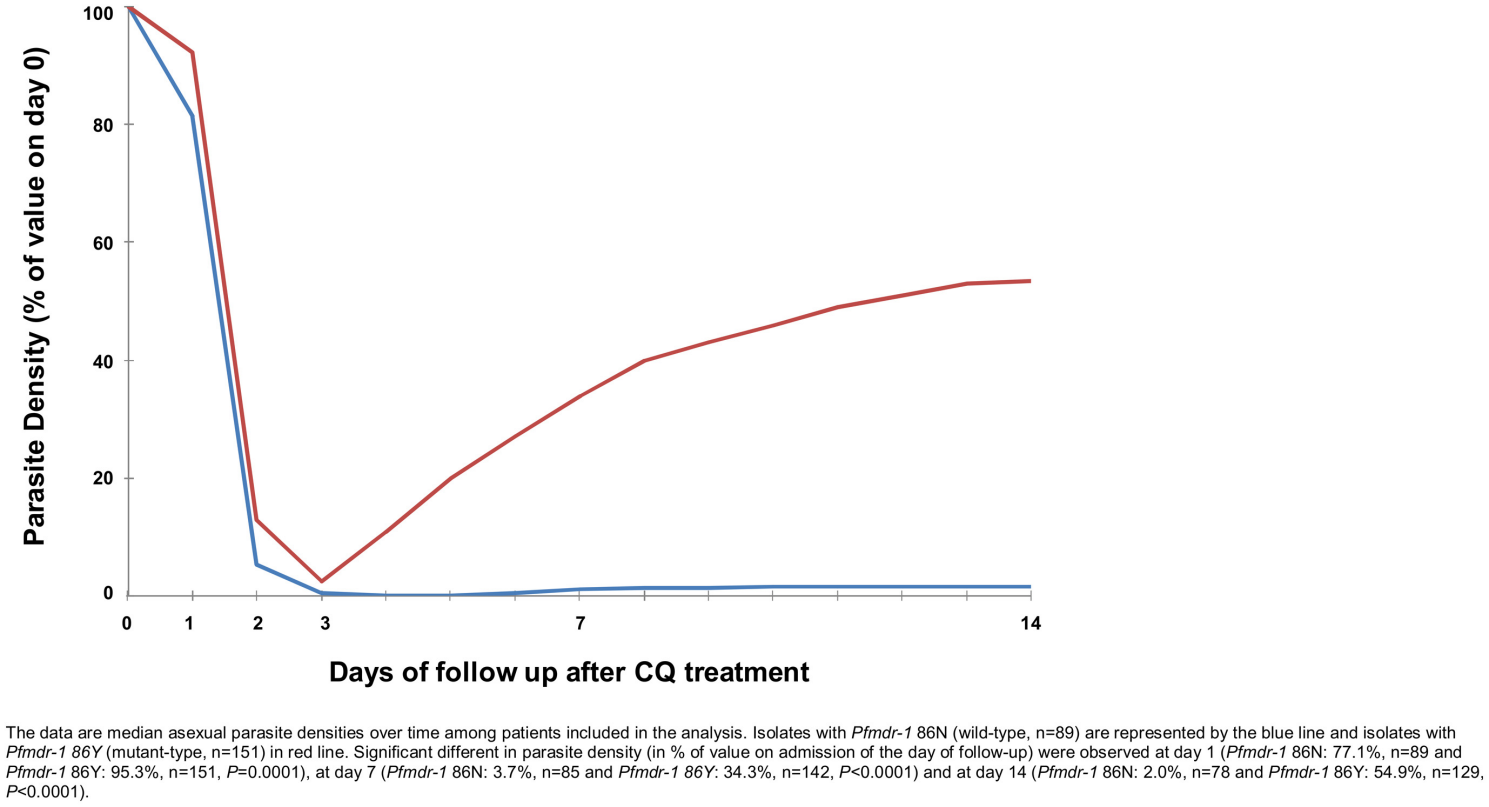
Parasites curves following CQ-treatment according *Pfmdr-1* alleles. Asexual parasites curves following CQ-treatment according *Pfmdr-1* mutation at position 86 in isolates from patients included in the analysis, Madagascar in 2006–2007.

Asexual parasite clearance following CQ-treatment displayed significant differences according to *Pfmdr-1* allelic form at position 86 at day 1 (*Pfmdr-1* 86N: 77.1%, n = 89 and *Pfmdr-1* 86Y: 95.3%, n = 151, P = 0.0001), day 7 (*Pfmdr-1* 86N: 3.7%, n = 85 and *Pfmdr-1* 86Y: 34.3%, n = 142, P<0.0001) and day 14 (*Pfmdr-1* 86N: 2.0%, n = 78 and *Pfmdr-1* 86Y: 54.9%, n = 129, P<0.0001) (Figure 3).

### Cumulative incidence of recurrence of patients and *Pfmdr-1* 86 mutation

**Figure 4:**
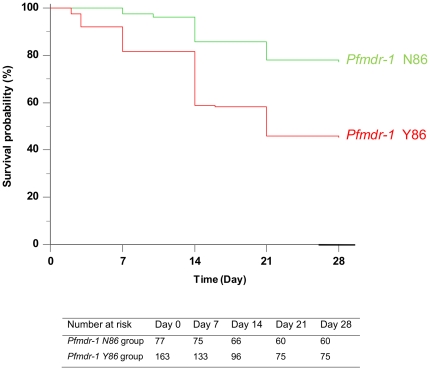
Curves of cumulative incidence of recurrence of patients over the 28-day follow-up period. Kaplan-Meier curves of cumulative incidence of recurrence of patients over the 28-day follow-up period according *Pfmdr-1* mutation at position 86 in isolates of day of recurrence from patients included in the analysis, Madagascar in 2006–2007.

The relation between *Pfmdr-1* 86Y mutation in isolates from day 0 (or day of recrudescence, if available) and clinical response to CQ treatment by using the time of recurrence, showed a markedly difference between the two curves (Figure 4). The cumulative incidence of recurrence of patients carrying the *Pfmdr-1* 86Y allele (n = 163, median time of recurrence 21 days) was significantly shorter than patients carrying *Pfmdr-1* 86N (n = 77, median time of recurrence 28 days). The log-rank test shows that the two curves differ significantly (*P*<0.0001) and the *Pfmdr-1* N86Y allele had a significant influence on the time of recurrence. The hazard ratio comparing the hazards in the two groups was estimated at 0.36 (95%IC: 0.23–055).

## Discussion

In the isolates from Madagascar studied here which harboured a wild type *Pfcrt*, *Pfmdr-1* polymorphism, in the absence of any noticeable gene amplification, seems to play a major role in late chloroquine clinical failures without affecting the overall level of *in vitro* CQ susceptibility. This situation contrasts with findings from multiple settings across the African continent, where CQ resistance depends primarily on mutations in *Pfcrt* gene and on additional mutations in *Pfmdr-1* gene, which may increase the level of resistance afforded by *Pfcrt*. *Pfcrt* mutant alleles seem universally found, albeit with different haplotypes in different geographical regions [22]. The association of *Pfmdr-1* with CQ resistance was not found in some areas [10], possibly reflecting the fact that the impact of the *Pfcrt/Pfmdr-1* combination mutation depends on the genetic background of the strain [23], [24] as demonstrated by studies with genetically manipulated lines or recombinant progeny of experimental crosses [23], [24] and the history of use of the antimalarial drugs (chloroquine and quinine being the two mains drugs used in Madagascar).

The observation that parasites with a wild *Pfcrt* allele were associated with a high rate of therapeutic failures of chloroquine is totally new. Several hypothesis may account for these findings. First, a particular *Pfcrt* haplotype, restricted to the Malagasy area and different from previously described resistant haplotypes, was present but not detected during this study. As we sequenced for all isolates a large part of *Pfcrt* gene including five codons consistently associated with CQ resistance in other settings, this hypothesis appears unlikely. It also is worth noting that so far the rare *Pfcrt* mutant alleles found in two sites from Madagascar (Andapa and Tsiroanomandidy) had a CVIET or CVIDT haplotype [25] and most likely were imported from the neighbouring Comoros Islands. Why did the CVIET haplotype not spread largely across Madagascar in spite of a significant drug pressure, as it did in Asia and Africa and even in the neighbouring Comoros Islands, remains to explain. A second hypothesis is that another gene, distinct from *Pfcrt* and not identified at present, is the main responsible for CQ resistance in Madagascar. Though this hypothesis cannot be formally ruled out, it appears also unlikely in view of the observed, strong association with *Pfmdr1*. A third possibility, is that the *Pfcrt* allele is present at a low fraction in the time 0 sample, too low to be detected (masked by the wild type allele and not detected by the PCR followed by DNA sequencing methodology) and too low to translate into a shifted IC_50_ *in vitro* (as these parasites represent a low fraction, they incorporate a low amount of 3H hypoxanthine and remain unnoticed). We also think this possibility unlikely, as no mutant *Pfcrt* could be detected in the samples collected from patients with late therapeutic failures. Indeed, the seminal studies by Djimde *et al.* in Mali showed that the minority CQ resistant clone selected during a treatment and causing a therapeutic failure accounted for a substantial proportion of the parasites on the day of recurrence [26]. Juliano *et al.* [27], using a nonradioactive heteroduplex tracking assay, reported recently that in a unique series of 17 patients in Madagascar, two (11.7%) harboured *Pfcrt* CVIET-resistant haplotype. The proportion of mutant parasites was 1.7 and 2.9% of the total parasite recurrent population obtained 14 days after the onset of CQ treatment, and neither of the two patients harboured any detectable CVIET parasites prior to treatment. In both cases the “recurrent” isolate 14 days after treatment by CQ contained a vast majority of CQ susceptible parasites (98.3% and 97.1% respectively), indicating that parasite genetic loci other than *Pfcrt* came into play in these recrudescences. We did not detect any *Pfcrt* resistant haplotype on the recurrence day of the 121 therapeutic failures of our series. Whether this reflects a somewhat lower sensitivity of our methodology compared to Juliano et *al* remains to be determined, but it is certainly consistent with the conclusion that these recrudescences are not associated with selection of a minority, mutant *Pfcrt* clone. The fourth hypothesis is that mutant alleles of *Pfmdr-1* play a major role in CQ clinical outcome. Ten years ago, transfection methods were used to explore the role of *Pfmdr-1* mutations in CQ resistance [28]. Introduction of wild-type polymorphisms into the resistant 7G8 line resulted in reduction of CQ resistance but introduction of mutations in the susceptible D10 line did not confer CQ resistance, which suggested that *Pfmdr-1* was not sufficient by itself to confer resistance. Lessons drawn from a recent genetic cross however indicate that observations with one or the other *P. falciparum* line are not directly applicable to all lines [24]. Different phenotypes of CQ resistance (and amodiaquine resistance) exist in the world, differing in different geographical areas. The contribution of *Pfmdr-1* mutations in CQ resistance varies depending on the particular *Pfcrt* haplotype with which they are associated [24].

The situation with regard to CQ resistance in Madagascar is unique. There were high rates of therapeutic failures, but those failures occurred in close to 90% of cases more than 7 days after treatment (Late Treatment Failures, LTFs) whereas in sub-Saharan Africa the proportion of LTFs is usually near 20% [29], [30], [31], [32], [33]. The proportion of isolates with detectable *Pfcrt* mutant parasites was totally disconnected from the rate of clinical failures. Moreover, no selection of *Pfcrt* mutant haplotypes between the first day of treatment and the day of recurrence was observed, which contrasted strongly with studies conducted in sub-Saharan countries [26]. We found also a selection of the N86Y or Y184F mutant alleles between the first day of treatment and the day of recurrence (10%), suggesting that *Pfmdr-1* has a major role in CQ clinical failures in Madagascar. However, this particular type of resistance was not reliably detected by the classical isotopic 48-h test used in our study, as only 3.3% of isolates showed phenotypic resistance (IC_50_>100 nM). The follow-up of circulating parasite densities over time in recurrent malaria episodes, showing rapid (but not complete) asexual parasites clearance within 72-hours following first day of treatment (Figure 3), was consistent with the apparent *in vitro* susceptibility of the isolates obtained in therapeutic failure cases. These results highlight the limits of the *in vitro* tests routinely used to monitor the antimalarial drug resistance. Based on the detection of the “trophozoites to schizonts” growth during a single parasite erythrocytic cycle, it appears that such *in vitro* assays can accurately detect high levels of resistance (corresponding usually to early treatment failures) but are unable to evaluate phenotypic resistance yielded by slow acting molecular mechanisms (or unusual mechanism), likely related to late failure treatments [23], [34].

The analysis of microsatellites flanking *Pfmdr-1* showed that mutations occurred on multiple genetic backgrounds (Figure 2). As previously described by Mehlotra et al. [9], we did not observe a strong reduction of heterozygosity in the CQ resistant parasite population, underlining that drug pressure from CQ treatment exerts a weaker selection on *Pfmdr-1* than on *Pfcrt*. This “soft” selection of *Pfmdr-1* mutant alleles may also be due to quinine pressure, as quinine was for a long time and until recently the antimalarial most commonly prescribed by practitioners in Madagascar [12]. The spread of *Pfmdr-1* mutant alleles may reflect a better fitness of parasites harbouring them or a better efficacy of the transmission [35].

Apart from the *Pfmdr-1*N86Y allele, the main risk factors associated with CQ therapeutic failures, lower age and lower prevalence of anti-PfMSP-1 antibodies, were inversely correlated with the immune status of patients. These results are similar with what has been reported for *Pfcrt* mutants in Mali and other countries where increasing age, reflecting the progressive acquisition of partial immunity, helped in the clearance of resistant parasites [26]. Hence, the immune status of patients probably explains why a significant proportion of patients having isolates harbouring a mutant *Pfmdr-1* allele were successfully cured by CQ in our series.

At present, CQ is being replaced by newer artemisinin-based combination drugs, such as artesunate-amodiaquine as first-line treatment in uncomplicated *falciparum* malaria in Madagascar. As *Pfmdr-1* is a major modulator of resistance to these drugs [10], monitoring in the future in which measure the high proportion of *Pfmdr-1* mutants observed in Madagascar can have an impact on the resistance of these antimalarials, even if at the moment this impact seems modest [11]. More generally, gaining insight about the mechanisms that regulate the genetic variation at *Pfmdr1* is important, particularly regarding the evolution and spread of *Pfmdr-1* alleles in *P. falciparum* populations under changing drug pressure which may have important consequences in terms of antimalarial use management.
